# Supplementary material for: clusterBMA: Bayesian model averaging for clustering
Source: PLoS One. 2023 Aug 21;18(8):e0288000. doi: 10.1371/journal.pone.0288000 (PMC10441802; doi:10.1371/journal.pone.0288000)
Supplement: S1 File — (PDF) [file pone.0288000.s001.pdf]

1 Supplementary Materials

1.1 Model Weights for Simulation Study 1

Cluster Separation	Algorithm	2 Dimensions	10 Dimensions	50 Dimensions
High	HC	0.099 (0.027)	0.038 (0.051)	0.004 (0.001)
	DIANA	0.105 (0.007)	0.108 (0.019)	0.123 (0.033)
	k-means	0.116 (0.003)	0.121 (0.007)	0.103 (0.014)
	PAM	0.116 (0.003)	0.124 (0.007)	0.170 (0.017)
	AP	0.116 (0.003)	0.121 (0.007)	0.102 (0.015)
	SC	0.114 (0.003)	0.123 (0.007)	0.160 (0.024)
	GMM	0.112 (0.002)	0.122 (0.007)	0.003 (0.001)
	SOM	0.108 (0.006)	0.120 (0.006)	0.165 (0.018)
	C-means	0.116 (0.003)	0.124 (0.007)	0.170 (0.017)
Medium	HC	0.079 (0.049)	0.003 (0.002)	0.008 (0.004)
	DIANA	0.079 (0.024)	0.111 (0.026)	0.108 (0.042)
	k-means	0.128 (0.011)	0.129 (0.006)	0.081 (0.020)
	PAM	0.128 (0.011)	0.138 (0.010)	0.193 (0.012)
	AP	0.128 (0.011)	0.126 (0.008)	0.082 (0.020)
	SC	0.102 (0.037)	0.117 (0.029)	0.196 (0.017)
	GMM	0.123 (0.010)	0.132 (0.009)	0.007 (0.004)
	SOM	0.106 (0.008)	0.107 (0.025)	0.128 (0.050)
	C-means	0.128 (0.011)	0.138 (0.010)	0.197 (0.019)
Low	HC	0.051 (0.042)	0.003 (0.001)	0.011 (0.004)
	DIANA	0.093 (0.021)	0.111 (0.022)	0.084 (0.042)
	k-means	0.132 (0.008)	0.124 (0.014)	0.094 (0.015)
	PAM	0.133 (0.009)	0.144 (0.011)	0.202 (0.016)
	AP	0.133 (0.008)	0.122 (0.012)	0.094 (0.016)
	SC	0.108 (0.029)	0.120 (0.028)	0.198 (0.013)
	GMM	0.120 (0.010)	0.128 (0.008)	0.010 (0.004)
	SOM	0.098 (0.027)	0.104 (0.028)	0.105 (0.034)
	C-means	0.131 (0.008)	0.144 (0.011)	0.202 (0.016)

Supplementary Table 1: Simulation Study 1: *clusterBMA* model weights - mean (SD) across each set of 10 simulated datasets. Model weights were calculated for 10 datasets in each combination of three clustering separation levels (Low, Medium and High) and differing number of dimensions (2, 10 and 50). HC = Hierarchical Clustering; DIANA = Divisive Analysis clustering; PAM = Partitioning Around Medoids; AP = Affinity Propagation; SC = Spectral Clustering; GMM = Gaussian Mixture Model; SOM = Self-Organising Map; C-means = Fuzzy C-means.

1.2 Additional Simulation Studies

To further demonstrate application of this approach, we conducted two additional simulation studies. The aim of the second simulation study was to investigate the effect of cluster separation on model-based uncertainty, and to test the utility of *clusterBMA* as ambiguity grows between sets of clustering results. In real world situations for applying unsupervised clustering, observed data will rarely be cleanly separated into well-defined clusters. In this section we aim to demonstrate the strength of our method for incorporating and accounting for the uncertainty that arises across multiple candidate models, when trying to identify cluster structure in data that may be representing overlapping or poorly separated groups.

The aim of the third simulation study was to demonstrate the ability of *clusterBMA* to average across models with differing numbers of clusters.

1.2.1 Simulation Study 2: Cluster Separation - Methods

We generated three simulated datasets using the R package clusterGenerate. This package allows the user to simulate data with multivariate normal clusters, and easily control the degree of separation between the clusters. Each simulated dataset contained 500 data points, with 100 points in each of 5 clusters. We generated a high separation dataset (separation value = 0.6), a medium separation dataset (separation value = 0.15), and a low separation dataset (separation value = 0). These separation values were chosen heuristically through trial and error, based on visual inspection of the plotted values.

For each simulated dataset we applied k-means, hierarchical clustering using Ward's method, and Gaussian mixture model, selecting the number of clusters $K_m = 5$ for each. Normalised weights $\hat{\mathcal{W}}_m$ were calculated to approximate the posterior model probability for each solution, based on Eq. 11 and 12. Each solution was represented as a pairwise similarity matrix, and these weights were used to calculate an element-wise weighted average across the three similarity matrices, resulting in a consensus matrix C. To calculate final probabilistic cluster allocations, we applied symmetric simplex matrix factorisation to the consensus matrix, using $K_{BMA} = 5$.

1.2.2 Simulation Study 2 - Results

Supplementary Figure 1 plots the simulated datasets. For each simulated dataset with high, medium and low separation between clusters, Supplementary Figures 2 - 4 present heatmaps for the similarity matrices from each algorithm with corresponding weights, and the consensus matrix generated by our pipeline.

Supplementary Figure 5 presents the final cluster allocations for each simulated dataset generated by *clusterBMA*, with point size scaled according to uncertainty of cluster allocation, where larger points representing higher uncertainty of allocation to a final cluster.

It is evident that as the degree of separation present between clusters in the data becomes lower, the degree of uncertainty in cluster allocations rises due to ambiguity and disagreement in clustering results across the multiple input algorithms. As real world data will typically not have clearly separated clusters, this demonstrates that in situations with messy data overlapping among possible clusters, it is valuable to use this Bayesian model averaging approach to take model-based uncertainty into account. Our method incorporates and quantifies this uncertainty, enabling cluster-based inferences that are better calibrated for this model-based source of uncertainty that is often ignored when using results from one chosen clustering algorithm.

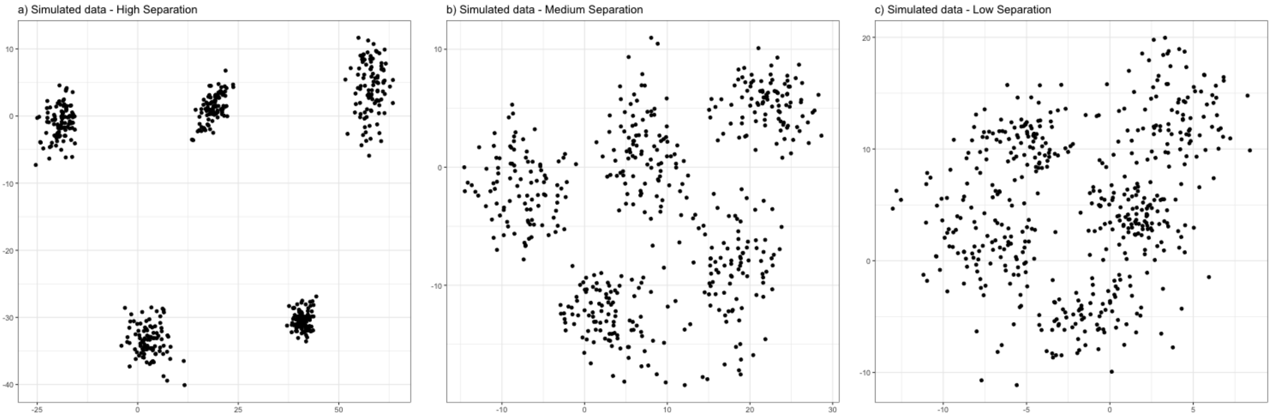

Supplementary Fig 1: Simulated datasets with 5 clusters - high, medium and low separation.

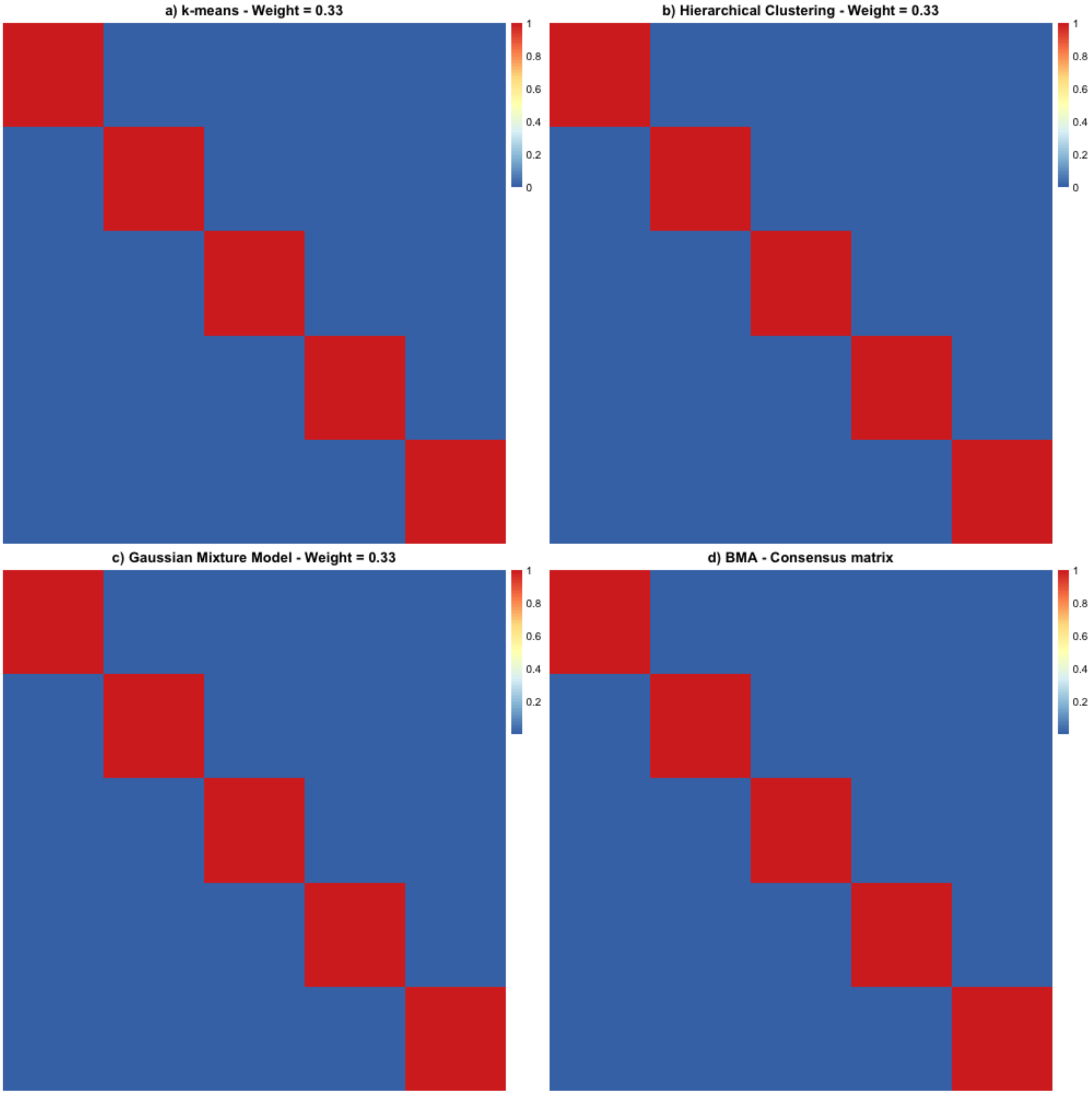

Supplementary Fig 2: High separation dataset - Heatmaps of similarity matrices for each clustering algorithm, and BMA Consensus matrix.

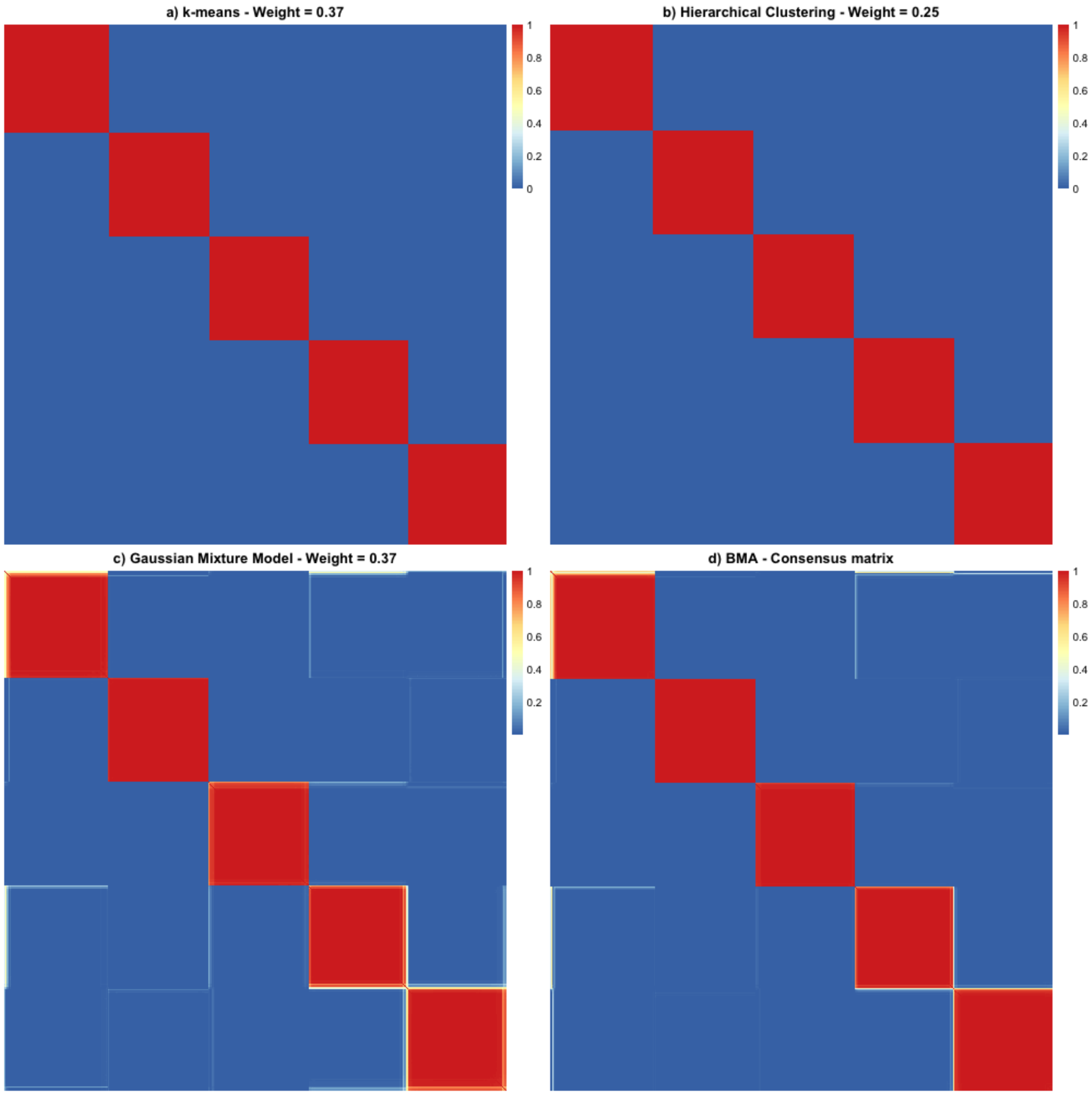

Supplementary Fig 3: Medium separation dataset - Heatmaps of similarity matrices for each clustering algorithm, and BMA Consensus matrix.

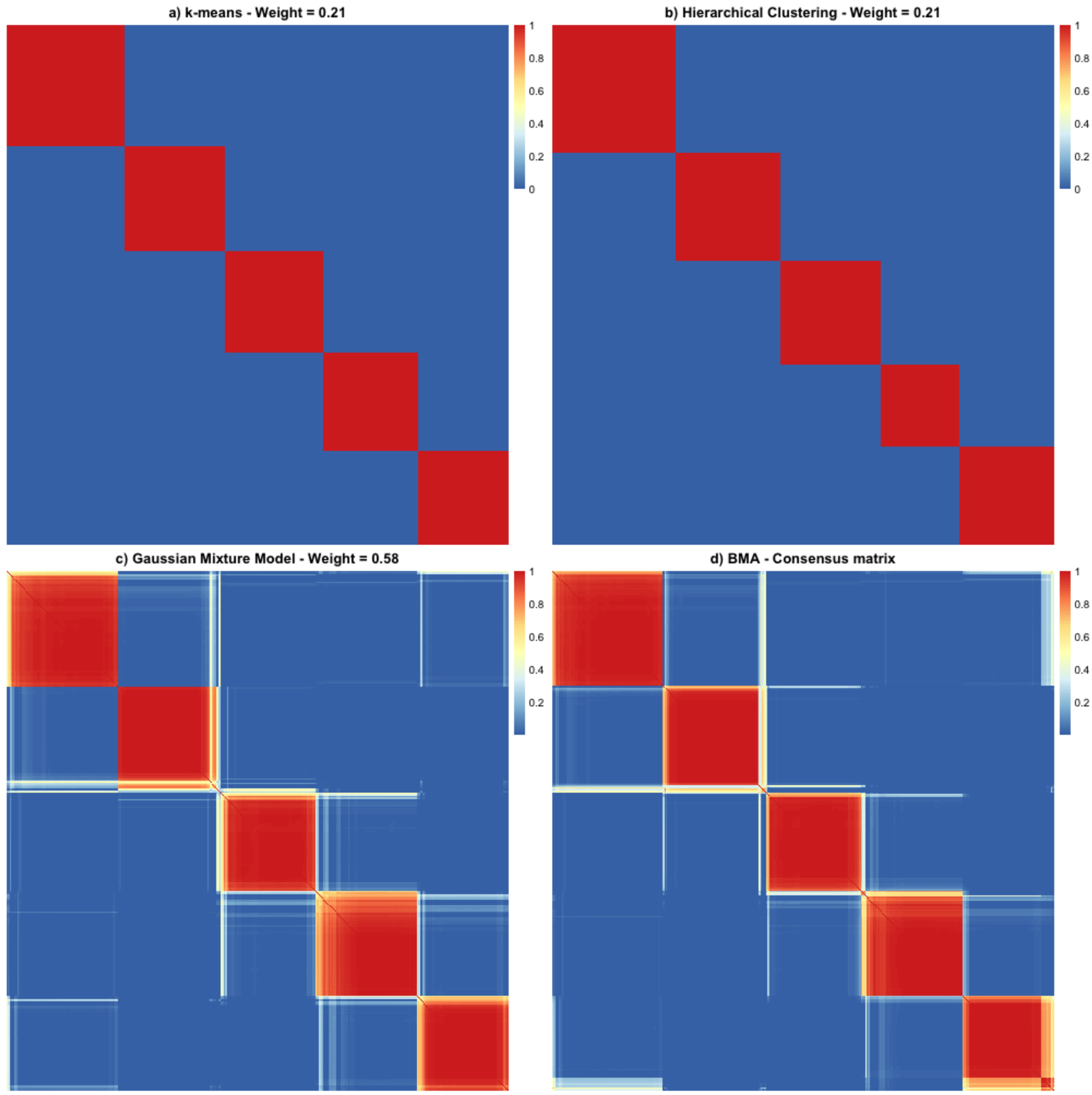

Supplementary Fig 4: Low separation dataset - Heatmaps of similarity matrices for each clustering algorithm, and BMA Consensus matrix.

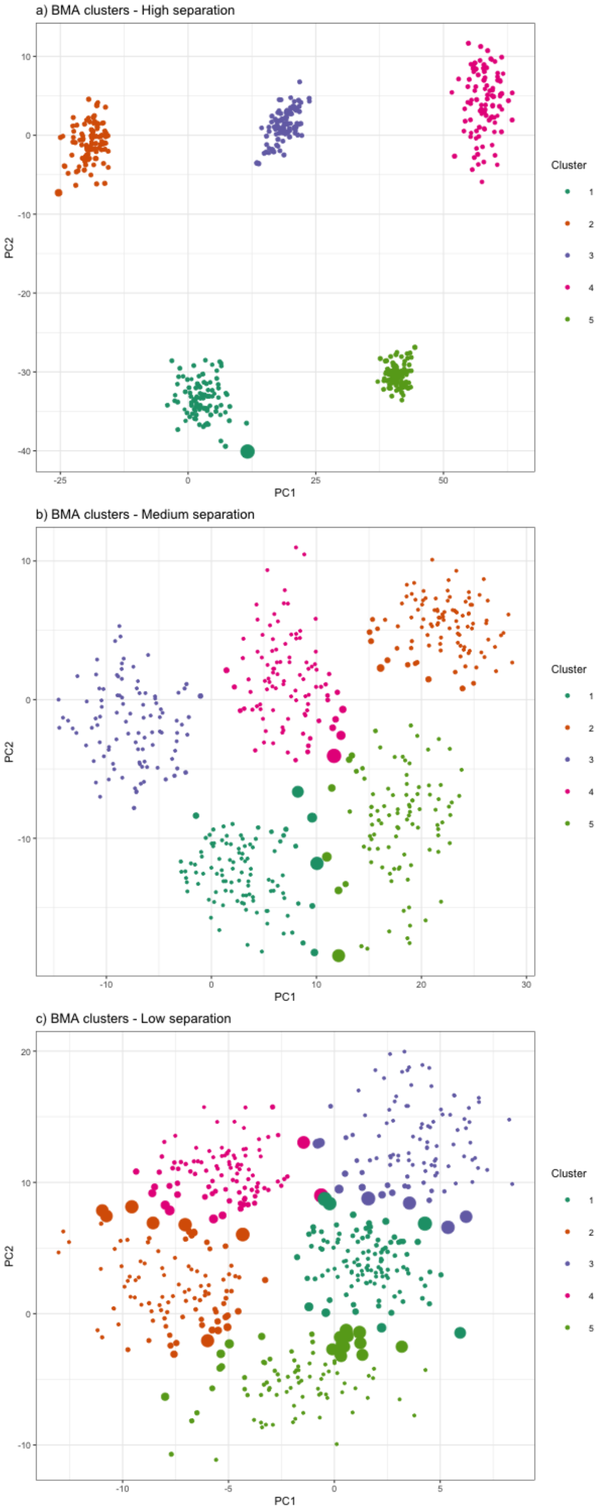

Supplementary Fig 5: Scatter plots of simulated datasets, coloured by final cluster membership. Point size is proportional to the uncertainty of cluster allocation, $p(\hat{g}_i \neq g_i)$. Larger points have greater uncertainty.

1.2.3 Simulation Study 3: Combining Results with Differing Numbers of Clusters - Methods

We generated one simulated dataset using the R package clusterGenerate. The simulated dataset contained 300 data points, with 100 points in each of 3 clusters, and a separation value of -0.3. We calculated two clustering solutions: k-means with K = 3, and HC with K = 2. As above, we applied the *clusterBMA* pipeline to combine results from these two models, with K_{BMA} set to 3.

1.2.4 Simulation Study 3 - Results

Supplementary Figure 6 presents the clustering allocations for k-means with (a) K = 3, (b) HC with K = 2, and (c) the *clusterBMA* combined solutions with $K_{BMA} = 3$ where larger points indicate greater allocation uncertainty. The normalised weights $\hat{\mathcal{W}}_m$ were 0.664 for the k-means solution, and 0.336 for the HC solution. From panel (c) in Supplementary Figure 6, there is low model-based uncertainty for cluster 1, moderate model-based uncertainty for clusters 2 and 3 where the algorithms disagree on the number of clusters for these points, and high model-based uncertainty at the border of cluster 2 with cluster 1 where the algorithms disagree on the allocation of marginal points. These results demonstrate that *clusterBMA* can combine clustering solutions across models with with differing numbers of clusters K_m.

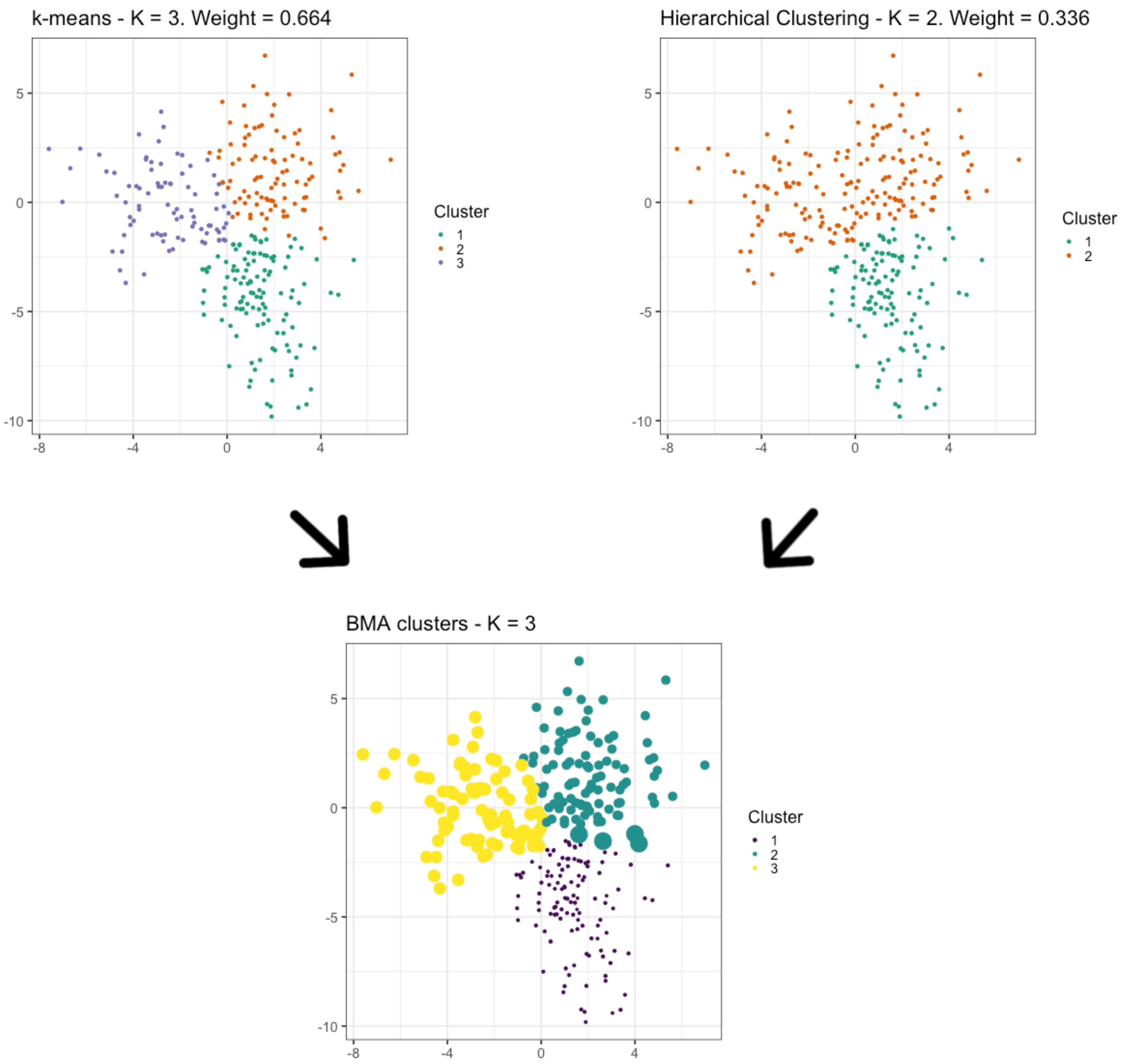

Supplementary Fig 6: Simulation Study 2: Combining solutions with differing numbers of clusters.
