# Supplementary material for: clusterBMA: Bayesian model averaging for clustering
Source: PLoS One. 2023 Aug 21;18(8):e0288000. doi: 10.1371/journal.pone.0288000 (PMC10441802; doi:10.1371/journal.pone.0288000)
Supplement: Attachment — Submitted filename: review.pdf [file pone.0288000.s002.pdf]

Review of paper ‘clusterBMA: Bayesian model averaging for clustering’

The authors have presented a new method for classification based on Bayesian model averaging. After reading the paper, I think that it still requires substantial work before it can eventually be published. Please, find some detailed comments below.

- Section 2.1. The method overview is fine but it comes too early in the paper, which makes it difficult to understand. Also, using A^m and S^m are a bit confusing and I would change it to A_m (as in K_m and other quantities that appear in the paper). Perhaps this section could be moved somewhere else in the paper.
- Section 2.2. The first sentence is a bit cryptic. I would include a description of what \Delta actually represents and provide some context.
- Section 2.2, eq (1). I believe that the left hand side should be p(\Delta = \Delta_m \par Y) as the right hand side involves \Delta_m.
- Section 2.2, eq. (2). are the probabilities in the right hand side correct? Why conditioning on i and j (in addition to M_m)? Also, I would expect the probability of two observations being in the same cluster to be computed using the joint posterior probabilities and not the marginal ones.
- Section 2.3, eq. (5). Although I can see the reason to use this approximation. How do the estimates of the effective number of parameters affect the estimates of P(M_m |Y)?
- Section 2.3, eq. (13). I see that that authors want (re-scaled) W_m to play the role of the marginal likelihood in their weighting scheme. However, is really the right hand side an approximation to the marginal likelihood? Is there a paper in which this approximation is actually developed and discussed?
- Sections 3.3 and 3.4. Are there tables similar to Table 2 for these scenarios? I have not found them in the Supplementary materials.
- Section 4. What is the number of features used in the analysis? In Section 4.2, it says “… 8 summary features in the frequency domain.” but then a dimension reduction is used. But the resulting dimension of the feature space is not mentioned at all.
- Section4. How fast is this method compared to others? Table 3 shows that GMM provides a very similar clustering as BMA (in terms of subjects per group).
- Figures 2 and 4 use colors for clusters 1 and 5 that make them difficult to identify.
- Also, the authors present a real example that can be tackled with standard methods. I would choose a different example in which their method ‘shines’ over existing methods.

Bibliography:

- Rusell et al. Has the wrong arXiv code as a dot is missing.
