# Supplementary material for: clusterBMA: Bayesian model averaging for clustering
Source: PLoS One. 2023 Aug 21;18(8):e0288000. doi: 10.1371/journal.pone.0288000 (PMC10441802; doi:10.1371/journal.pone.0288000)
Supplement: Attachment — Submitted filename: Reponse_to_Reviewers_pt2.pdf [file pone.0288000.s004.pdf]

Response to Reviewers comments

Title: clusterBMA: Bayesian model averaging for clustering

Thank you for reviewing our paper. I have attached the revised paper and have outlined below how the paper has been modified to address the comments of the editor, and Reviewer #2.

<u>Editor's Comments</u>

Editor: I agree with reviewer #2's comment regarding Section 2.1. Since many symbols from Section 2.1 are introduced in subsequent sections, I propose moving Section 2.1 after Section 2.4.

Reviewer #2: I still think that Section 2.1 should be moved from there. As it is now, the only option for the reader is to skip it on a first read. Hence, there is no point in keeping it where it is. However, I'll leave that decision to the editor.

Thank you both for your comments. We have made the suggested change in the attached manuscript, and moved the overview to section 2.4.

Kind Regards,
Owen Forbes (on behalf of the co-authors)
